# Medication use in a large international sample of people with multiple sclerosis: associations with quality of life, relapse rate and disability

**DOI:** 10.1179/1743132815Y.0000000036

**Published:** 2015-08

**Authors:** George A. Jelinek, Tracey J. Weiland, Emily J. Hadgkiss, Claudia H. Marck, Naresh Pereira, Dania M. van der Meer

**Affiliations:** 1 Emergency Practice Innovation Centre, St Vincent's Hospital, Melbourne, Victoria, Australia; 2 Department of Epidemiology and Preventive Medicine, Monash University, Melbourne, Victoria, Australia; 3 Department of Medicine, The University of Melbourne, St Vincent's Hospital, Melbourne, Victoria, Australia; 4 Department of Medicine, Box Hill Hospital, Melbourne, Victoria, Australia

**Keywords:** Multiple sclerosis, Disease-modifying drugs, Medications, Health-related quality of life, Relapse rate, Disability

## Abstract

**Objectives:**

To examine associations between medication use and health-related quality of life (HRQOL), relapse rate and disability in an international cohort of people with multiple sclerosis (PwMS).

**Methods:**

Using Web 2.0 platforms, the authors recruited PwMS who completed survey items on demographics, medication use, HRQOL, relapse rate and disability.

**Results:**

Of 2276 respondents from 56 countries, approximately half were taking a disease-modifying drug (DMD), most commonly glatiramer acetate or an interferon. Use of DMDs was not consistently associated with HRQOL. Individually, glatiramer acetate was associated with better HRQOL when compared with other DMDs or no DMD use. Overall, DMD use was neither associated with disability nor lower relapse rate, although those taking a DMD >12 months had 23.9% fewer relapses than those not taking a DMD. Polypharmacy, defined as those taking five or more over the counter, prescription or herbal medications, irrespective of DMD use, was associated with markedly worse HRQOL across all domains.

**Discussion:**

There was no consistent association of DMD use with better health outcomes in this large international \sample of PwMS, although relapse rate appears lower for those taking a DMD for >12 months. Glatiramer acetate had associations with better HRQOL compared with other DMDs.

## Introduction

Over the last two decades, a large number of medications has been researched and approved for the management of multiple sclerosis (MS).^1^ Currently at least 10 medications are licensed for use around the world. All have been shown in randomized controlled phase III trials to have a significant short term effect in reducing the rate of relapses for people with relapsing-remitting MS (RRMS), the most common form of the disease. However, long term benefit with respect to accumulation of disability has been more difficult to demonstrate and few of the medications appear to have any significant effect on progressive forms of the disease.^1^ Although mostly well tolerated, the likelihood of long term adherence to these medications is limited by a number of side effects, some serious. There is a need for more research on use of the disease-modifying drugs (DMDs) for MS outside of clinical trials to get a better sense of their efficacy in real-world situations.

The first generation DMDs were released in the 1990s. Comprising three self-injected interferon beta medications and glatiramer acetate, these drugs were shown in clinical trials to result in a modest but significant reduction in relapse rate of ∼30%, with little effect on disability. Their safety profiles were considered acceptable, although the interferons had significantly more systemic side effects than glatiramer acetate, whose side effects were largely limited to localized skin reactions. The interferons were injected second daily (Betaferon or Betaseron), thrice weekly (Rebif), or weekly (Avonex), whereas glatiramer acetate (Copaxone) was initially shown to be effective with daily administration. More recently, it has been shown to be effective with and licensed for second daily injection at double the original dose.^2^

Second generation DMDs include the monoclonal antibody natalizumab (Tysabri), delivered by monthly intravenous infusion, and associated medications including the recently licensed alemtuzumab (Lemtrada) and others still under investigation such as daclizumab (Zenapax) and rituximab (MabThera). A number of oral drugs has also recently been approved including fingolimod (Gilenya), dimethyl fumarate (Tecfidera) and teriflunomide (Aubagio), while oral cladribine (Movectro) has been withdrawn following difficulties in licensing and laquinimod is still under investigation. Fingolimod and teriflunomide are taken once daily, and dimethyl fumarate twice daily.

These second generation DMDs in general have also been shown to reduce relapse rates, however, because study cohorts were entirely different and relapse rates were higher when the first generation drugs were tested, in addition to a paucity of head-to-head trials, it is not possible to make inferences about comparative efficacy; in general they do, however, have potentially more serious side effects. An effect on disability progression has remained difficult to demonstrate.

Other general immunosuppressant medications have also been used in MS, with varying degrees of efficacy. Of these, only mitoxantrone (Novantrone) has been approved for use in rapidly progressive MS and is the only agent generally approved for use in secondary progressive MS (SPMS), although interferon B-1b has been approved for this indication in Europe. While highly effective in stabilizing the disease, serious side effects including cardiomyopathy (even with low dose^3^) and treatment-induced leukaemia have limited its use.^4^

While the advent of these medications for the management of MS has been described as one of the most rapidly advancing areas in neurological research,^5^ peak regulatory bodies have cast doubt on the cost effectiveness of the first generation DMDs, with the National Institute for Health and Clinical Excellence (NICE) in the UK rating them the least cost effective of all marketed pharmaceuticals between 1996 and 2005.^6^

As part of the Health Outcomes and Lifestyle Interventions in a Sample of people with Multiple sclerosis (HOLISM) study,^7^ the authors collected data on medication use from this cohort of ∼2000 people with MS worldwide, with the aims of reporting current patterns of medication use and detecting associations between medication use and the disease outcomes, relapse rate, disability and health-related quality of life (HRQOL).

## MATERIALS AND METHODS

### Design and procedures

The methodology of the HOLISM study has been reported in detail elsewhere.^7^ In short, participants aged ≥ 18 years, with a definite diagnosis of MS made by a physician, were recruited between May and September 2012 via Web 2.0 platforms, including social media groups, websites, forums and MS society sites. They were given written information and provided consent to undertake a comprehensive online cross-sectional survey of ∼45 minutes duration. Ethics approval was granted by St Vincent's Hospital Research Ethics Committee (LRR 055/12).

### Tools used

The survey used validated tools where possible to assess MS disease outcomes including HRQOL using the multiple sclerosis quality of life-54 (MSQOL-54), disability using the patient-determined disease steps (PDDS), and self-reported number of doctor-diagnosed relapses over the last 12 months. Participants reported current and previous use of medications for MS, including length of time taken, for 24 listed DMDs and other medications commonly taken by people with multiple sclerosis (PwMS) (including generic and trade names). Participants were also asked whether they took prescription, over-the-counter, or herbal agents for 10 symptomatic conditions: depression, anxiety, headaches, other pain, fatigue, difficulty sleeping, bladder problems, bowel problems, spasticity and ‘other’. These may have included medications already reported under the previous list of agents, in particular baclofen for spasticity. The authors analysed these data both descriptively in terms of number of agents taken and also compared those with polypharmacy ( ≥ 5 agents) versus those without in terms of HRQOL, relapse rate and disability.

The MSQOL-54 is a measure of HRQOL developed from the RAND 36-Item Health Survey (SF-36) supplemented with 18 additional items. The MSQOL-54 has 52 items distributed into 12 scales, and two single items, producing physical and mental health composite scores. The tool has been extensively validated and translated in international populations,^8^–^10^ and in assessing the impact of fatigue,^11^ depression^12^ and sexual dysfunction,^13^ as well as a number of medical therapies. The MSQOL-54 was scored according to the scoring instructions with a set number of items required to be completed in order to give rise to the subscores, which in turn were required for calculation of the composite scores; hence there was variability in the completion rates.

The PDDS is a self-reported surrogate tool for the expanded disability status scale (EDSS) which is commonly used by neurologists to assess gait disability.^14^ It is scored ordinally from 0 (normal) to 8 (bed bound). It correlates well with EDSS and has excellent concordance between raters. It is a practical tool to use to assess changes in disability over time.^15^ The PDDS has been used in a number of studies associated with the NARCOMS registry.^16^–^18^ The fatigue severity scale (FSS) was used to assess clinically significant fatigue with a mean score >3 taken as the cut-off.^19^

### Data analysis

The Statistical Package for the Social Sciences (SPSS) version 20.0 was used to calculate statistics. Medications used were grouped into the categories outlined in the Introduction, plus the groups ‘Steroids’ and ‘Other’ (Table 1). Free text responses for ‘other’ medications were reviewed and re-categorized into existing variables, being manually recoded where the medication had been listed but the respondent had apparently failed to recognize the name or overlooked it. Summary scores from validated tools were derived according to scoring instructions or as suggested in the literature. The authors collapsed the PDDS from nine categories into three (normal, mild, moderate disability = ‘normal/some disability’; gait disturbance, cane, late cane = ‘moderate disability’; bilateral support, wheelchair, bedridden = ‘major disability’).

**Table 1:** Number (%) of people with MS currently taking each medication, duration and number (%) previously taking each medication

Class of medication	Medication name	Number currently taking	Time taken (years)**				Number previously taking
		n = 2276	< 1 year	1–10 years	10+ years	Missing*	*n* = 2276
First generation DMDs	Glatiramer acetate	488 (21.4)	125 (25.6)	321 (65.8)	25 (5.1)	17 (3.5)	345 (15.2)
	Interferons	433 (19.0)	100 (23.1)	266 (61.4)	51 (11.8)	16 (3.7)	570 (25.0)
Second generation DMDs	Natalizumab	132 (5.8)	56 (42.4)	70 (53.0)	1 (0.8)	5 (3.8)	77 (3.4)
	Alemtuzumab	2 (0.1)	2 (100)	0 (0)	0 (0)	0 (0)	3 (0.1)
	Daclizumab	1 (0.1)	1 (100)	0 (0)	0 (0)	0 (0)	2 (0.1)
	Rituximab	2 (0.1)	1 (50.0)	1 (50.0)	0 (0)	0 (0)	5 (0.2)
	Fingolimod	94 (4.1)	71 (75.5)	22 (23.4)	0 (0)	1 (1.1)	25 (1.1)
	Dimethyl fumarate	22 (1.0)	9 (40.9)	11 (50.0)	0 (0)	2 (9.1)	7 (0.3)
	Teriflunomide	4 (0.2)	0 (0)	3 (75.0)	0 (0)	1 (25.0)	0 (0)
	Cladribine	2 (0.1)	2 (100)	0 (0)	0 (0)	0 (0)	0 (0)
	Laquinimod	3 (0.1)	0 (0)	3 (0.1)	0 (0)	0 (0)	2 (0.1)
General immunosuppressants	Azathioprine	7 (0.3)	6 (85.7)	1 (14.3)	0 (0)	0 (0)	22 (1.0)
	Cyclophosphamide	1 (0.1)	1 (100)	0 (0)	0 (0)	0 (0)	14 (0.6)
	Methotrexate	5 (0.2)	2 (40.0)	3 (60.0)	0 (0)	0 (0)	26 (1.1)
	Mitoxantrone	4 (0.2)	3 (75.0)	0 (0)	0 (0)	1 (25.0)	45 (2.0)
	Mycophenolate	5 (0.2)	2 (40.0)	3 (60.0)	0 (0)	0 (0)	3 (0.1)
Steroids	Adrenocorticotropic hormone (ACTH)	9 (0.4)	2 (22.2)	0 (0)	0 (0)	7 (77.8)	28 (1.2)
	Prednisolone	144 (6.3)	99 (68.7)	15 (10.4)	7 (4.9)	23 (16.0)	756 (33.2)
Others	Immunoglobulins	5 (0.2)	1 (20.0)	4 (80.0)	0 (0)	0 (0)	23 (1.0)
	Plasmapheresis	0 (0)	0 (0)	0 (0)	0 (0)	0 (0)	11 (0.5)
	LDN	163 (7.2)	44 (27.0)	111 (68.1)	3 (1.8)	5 (3.1)	67 (2.9)
	Minocycline	16 (0.7)	9 (56.2)	7 (43.8)	0 (0)	0 (0)	13 (0.6)
	Baclofen	242 (10.6)	57 (23.6)	147 (60.7)	29 (12.0)	9 (3.7)	127 (5.6)
	Fampridine	80 (3.5)	40 (50.0)	36 (45.0)	1 (1.2)	3 (3.8)	36 (1.6)

DMD: disease-modifying drug; Low-dose naltrexone; MS: multiple sclerosis.

* Data for time taking medication not provided by respondent

** Denominator is variable based on number currently taking specific medication.

The authors report number (%) for medication use. Percentages reported were calculated using the total completing the medication section as the denominator. Bivariate analyses of continuous data were performed using independent samples *t*-test for comparisons of two groups, or analysis of variance (ANOVA) for comparisons of three groups, with Bonferroni adjustments for multiple comparisons and means (95% CI) reported. Categorical data were compared using Pearson's chi square for comparisons involving more than two groups and adjusted standardized residuals (>2.0 and < -2.0) were used to indicate over and under-representation. For all inferential analyses, the authors report two-tailed tests of significance with alpha set at 0.05.

Relapse rates, based on number of self-reported doctor-diagnosed relapses, were compared not only for those people with RRMS taking a DMD versus those not taking, but also in a pre-planned analysis, for a subset of participants taking a DMD for >12 months. A 12-month period was chosen from starting the DMD to more clearly reflect relapse rate for the group when stable on medication. Health-related quality of life for the four most frequently used DMDs (glatiramer acetate, interferons, fingolimod and natalizumab) was compared against HRQOL for those using all other DMD apart from that DMD and those not using DMDs. Four domains of HRQOL were used to illustrate the associations of each DMD with HRQOL: overall quality of life (QOL), physical health composite, mental health composite and health perception. In addition, as low-dose naltrexone (LDN) is often used as a DMD by PwMS, although not licensed for such use, HRQOL was similarly compared for this medication.

The crude and adjusted unstandardized regression coefficient for mental health composite HRQOL and physical health composite HRQOL, associated with DMD use were assessed using linear regression and multiple linear regression. The relationship between 10 potential covariates (age, gender, marital status, years since diagnosis, number of children, employment status, education, number of close relationships, disability and number of comorbidities) and the outcomes of interest, either mental health composite or physical health composite, were assessed in a series of separate regressions. Variables showing a significant (*P* < 0.05) association with the outcome were retained as covariates. Variables were excluded as covariates if they showed a strong relationship with other potential covariates or if variance inflation factor exceeded 5. Other assumptions tested included normality, linearity, and homoscedasticity which were assessed by visual inspection of histograms and residual plots (plots of the standardized residuals as a function of standardized predicted values). For both physical health composite and mental health composite, adjustments were made for age, marital status, number of children, employment status, education, number of close relationships, disability and number of comorbidities).

## Results

### Demographics

Overall, among participants responding to the medication questions, with denominators adjusted for missing data, 1855 (82.4%) were women and 396 (17.6%) men. Most (715, 32.3%) were aged 40–49 years, followed by 589 (26.6%) 50–59 years, 565 (25.5%) 30–39 years, 228 (10.3%) ≥ 60 years and 116 (5.2%) 17–29 years. Residents of the USA, Australia, UK, New Zealand and Canada comprised 88% of the HOLISM study sample. Over half (1209, 53.8%) had a normal BMI according to the WHO definition,^20^ while 515 (22.9%) were overweight, 431 (19.2%) obese and 94 (4.2%) underweight. The great majority (1675, 74.6%) were married or partnered, 314 (14.0%) were single, 230 (10.2%) separated or divorced and 26 (1.2%) widowed, and the majority (1544, 68.7%) had children.

Many (744, 32.8%) worked full-time, with 486 (21.4%) part-time, although 521 (22.9%) had retired because of disability and 71 (3.1%) because of age; there were 173 (7.6%) PwMS who were stay at home with parents or carers and the same number unemployed; others, including students, comprised 103 people (4.5%). With respect to time from diagnosis, the largest group were fairly recently (up to 5 years) diagnosed (1028, 45.3%), followed by those diagnosed 6–10 years (529, 23.3%), 11–15 years (361, 15.9%), 16–20 years (173, 7.6%), 21–25 years (89, 3.9%), and 87 (3.8%) people diagnosed longer, 3 of them for over 40 years.

### Medication use

Overall, of 2276 respondents answering relevant questions related to medication use, 752 (33.0%) had never taken one of the 24 listed DMDs, 384 (16.9%) had previously used a DMD but had discontinued use and were no longer taking a DMD, 421 (18.5%) had previously taken a DMD but had discontinued it and were now taking another DMD, while 719 (31.6%) were currently taking a DMD having not taken one previously. Of those currently taking a DMD, 1103 (98.6%) reported taking one DMD only, with 37 (1.4%) taking more than one DMD. Of these taking more than one DMD, most (29/37) reported taking an interferon as well as another drug or drugs. These 29 people were also taking glatiramer acetate in 15 cases, natalizumab in 10 cases, dimethyl fumarate in 5 cases, fingolimod in 4 cases and teriflunomide in 1 case.

The number of respondents taking each of the medications listed in the survey, as well as the duration, is reported in Table 1, along with the number who had previously taken each of the medications. Table 2 reports the number of respondents taking each of the medications listed in the survey by disease type.

**Table 2:** Medications taken by disease type

Class of medication	Medication name	RRMS	PPMS	SPMS	PRMS	BMS	Unknown MS type	Missing*	Total
First generation DMDS	Glatiramer acetate	384 (78.7)	10 (2.0)	6 (1.2)	6 (1.2)	8 (1.6)	52 (10.7)	22 (4.5)	488 (100)
	Interferons	338 (78.1)	7 (1.6)	5 (1.2)	5 (1.2)	8 (1.8)	44 (10.2)	26 (6.0)	433 (100)
Second generation DMDs	Natalizumab	103 (78.0)	6 (4.5)	7 (5.3)	7 (5.3)	0 (0)	8 (6.0)	1 (0.8)	132 (100)
	Alemtuzumab	0 (0)	0 (0)	0 (0)	0 (0)	0 (0)	0 (0)	2 (0.0)	2 (100)
	Daclizumab	0 (0)	0 (0)	0 (0)	0 (0)	0 (0)	0 (0)	1 (100)	1 (100)
	Rituximab	2 (100.0)	0 (0)	0 (0)	0 (0)	0 (0)	0 (0)	0 (0)	2 (100)
	Fingolimod	67 (71.3)	6 (6.4)	1 (1.1)	1 (1.1)	1 (1.1)	8 (8.5)	10 (10.6)	94 (100)
	Dimethyl fumarate	11 (50.0)	1 (4.5)	1 (4.5)	1 (4.5)	1 (4.5)	3 (27.2)	4 (18.2)	22 (100)
	Teriflunomide	3 (75.0)	0 (0)	0 (0)	0 (0)	0 (0)	1 (25.0)	0 (0)	4 (100)
	Cladribine	1 (50.0)	0 (0)	0 (0)	0 (0)	0 (0)	0 (0)	1 (50.0)	2 (100)
	Laquinimod	3 (100.0)	0 (0)	0 (0)	0 (0)	0 (0)	0 (0)	0 (0)	3 (100)
General immunosuppressants	Azathioprine	1 (14.3)	1 (14.3)	0 (0)	0 (0)	0 (0)	1 (14.3)	4 (57.1)	7 (100)
	Cyclophosphamide	0 (0)	1 (100.0)	0 (0)	0 (0)	0 (0)	0 (0)	0 (0)	1 (100)
	Methotrexate	3 (60.0)	1 (20.0)	0 (0)	1 (20.0)	0 (0)	0 (0)	0 (0)	5 (100)
	Mitoxantrone	1 (25.0)	1 (25.0)	0 (0)	0 (0)	0 (0)	1 (25.0)	1 (25.0)	4 (100)
	Mycophenolate	1 (20.0)	2 (40.0)	0 (0)	1 (20.0)	1 (20.0)	0 (0)	0 (0)	5 (100)
Steroids	Adrenocorticotropic hormone (ACTH)	3 (33.3)	1 (1.1)	1 (1.1)	0 (0)	0 (0)	3 (33.3)	1 (1.1)	9 (100)
	Prednisolone	94 (65.3)	12 (8.3)	3 (2.1)	3 (2.1	3 (2.1)	19 (13.2)	10 (6.9)	144 (100)
Others	Immunoglobulins	2 (40.0)	2 (40.0)	0 (0)	0 (0)	0 (0)	0 (0)	1 (20.0)	5 (100)
	Plasmapheresis	0 (0)	0 (0)	0 (0)	0 (0)	0 (0)	0 (0)	0 (0)	0 (100)
	LDN	72 (44.2)	23 (14.1)	3 (1.8)	3 (1.8)	1 (0.6)	26 (3.7)	35 (21.5)	163 (100)
	Minocycline	11 (68.8)	1 (6.3)	0 (0)	0 (0)	0 (0)	4 (25.0)	0 (0)	16 (100)
	Baclofen	91 (37.6)	38 (15.7)	12 (5.0)	12 (5.0)	2 (0.8)	25 (10.3)	62 (0.1)	242 (100)
	Fampridine	30 (37.5)	11 (13.8)	5 (6.3)	5 (6.3)	0 (0)	14 (17.5)	15 (18.8)	80 (100)

DMD: disease-modifying drug; RRMS: relapsing-remitting MS; PPMS: primary progressive MS; SPMS: secondary progressive MS; PRMS: progressive relapsing MS; BMS: benign MS; LDN: low-dose naltrexone.

* Data for type of MS not provided by respondent.

#### Over the counter, prescription and herbal agents

The number of participants taking over the counter, prescription and herbal agents for the listed complaints is tabulated in Table 3. Many participants were taking several of these agents for various complaints, with 426 (18.7%) participants taking one agent, 374 (16.4%) taking two, 288 (12.7%) taking three, 184 (8.1%) taking four, 135 (5.9%) taking five, 103 (4.5%) taking six, and 102 (4.5%) taking seven or more, up to a maximum of 14 agents for one participant. A total of 664 (29.2%) participants did not report taking any agent. Similar rates of polypharmacy were present for those taking a DMD for ≥ 12 months, and those not taking a DMD (117/732, 16.0% vs 154/1136, 13.6%, *P* = .158).

**Table 3:** Number of participants taking other over the counter, prescription and herbal agents by complaint for which taken (*n* = 2276)

Condition	Over the counter	Prescription	Herbal	Total participants taking a treatment*
Pain	462 (20.3)	389 (17.1)	66 (2.9)	826 (36.3)
Headaches	650 (28.6)	195 (8.6)	40 (1.8)	817 (35.9)
Depression	9 (0.4)	502 (22.1)	69 (3.0)	556 (25.0)
Sleep difficulties	131 (5.8)	292 (12.8)	170 (7.5)	552 (24.3)
Spasticity	32 (1.4)	363 (15.9)	76 (3.3)	453 (19.9)
Fatigue	48 (2.1)	236 (10.4)	134 (5.9)	401 (17.6)
Bladder problems	27 (1.2)	306 (13.6)	74 (3.3)	391 (17.2)
Anxiety	11 (0.5)	258 (11.3)	99 (4.3)	358 (15.7)
Bowel problems	128 (5.6)	102 (4.5)	85 (3.7)	290 (12.7)
Other	33 (1.4)	121 (5.3)	78 (3.4)	215 (9.4)

Values in brackets refer to the percentage of participants overall taking that category of agent for a particular complaint

* Many were taking agents from more than one category.

### Health outcomes

As the use of DMDs in combination is not currently recommended and there are no available data on outcomes of such combinations,^21^ and because of the small numbers in that subgroup limiting meaningful analysis, the associations of HRQOL, relapse rate and disability were explored only for those taking a single DMD.

#### Health-related quality of life

Health-related quality of life was not consistently associated with DMD use or non-use depending on domain. The HRQOL was statistically significantly higher for those taking a single DMD in the physical health and role limitations physical subscores, but was significantly better for those not taking a DMD in mental health composite, emotional wellbeing, cognitive function and health distress subscores (Table 4). The magnitude of the difference was generally small and probably clinically insignificant except for physical health subscore, at a 7.6-point difference, given that HRQOL measurements are derived from the SF-36 and it is generally accepted that an improvement in this scale of five points is clinically significant.^22^,^23^

**Table 4:** HRQOL composites and subscores by use of a single DMD or not*

HRQOL composite/subscore	Single DMD or no DMD	*n*	Mean HRQOL	SD	*P*
Overall QOL subscore	No DMD	1104	66.8	19.4	0.472
	DMD	1081	67.4	18.7	
Physical health composite	No DMD	919	59.0	22.1	0**.**403
	DMD	958	59.8	20.8	
Mental health composite	No DMD	1078	68.2	21.0	**0.011**
	DMD	1061	65.9	21.6	
Physical health subscore	No DMD	1125	56.4	34.9	**0.000**
	DMD	1096	64.0	31.1	
Role limitations physical subscore	No DMD	1123	44.3	43.3	**0.049**
	DMD	1092	47.9	43.5	
Role limitations emotional subscore	No DMD	1117	69.7	41.0	0.373
	DMD	1080	68.1	41.3	
Pain subscore	No DMD	1129	72.0	26.2	0.985
	DMD	1090	72.0	26.1	
Emotional wellbeing subscore	No DMD	1132	70.1	18.2	**0.001**
	DMD	1098	67.4	19.1	
Energy subscore	No DMD	1132	44.5	23.1	0.121
	DMD	1098	43.0	22.0	
Health perception subscore	No DMD	1130	57.2	23.4	0.082
	DMD	1097	55.5	21.9	
Social function subscore	No DMD	1094	69.0	24.9	0**.**118
	DMD	1067	70.6	23.2	
Cognitive function subscore	No DMD	1132	68.2	26.3	**0.002**
	DMD	1098	64.6	27.3	
Health distress subscore	No DMD	1130	61.8	27.0	**0.009**
	DMD	1098	58.8	27.3	
Sexual function subscore	No DMD	976	65.4	31.3	0.113
	DMD	999	67.6	30.0	

HRQOL: health-related quality of life; DMD: disease-modifying drug.

* Data are for respondents taking one DMD only. Bold denotes statistically significant.

#### Health-related quality of life with individual DMDs and LDN

**Figure 1.**
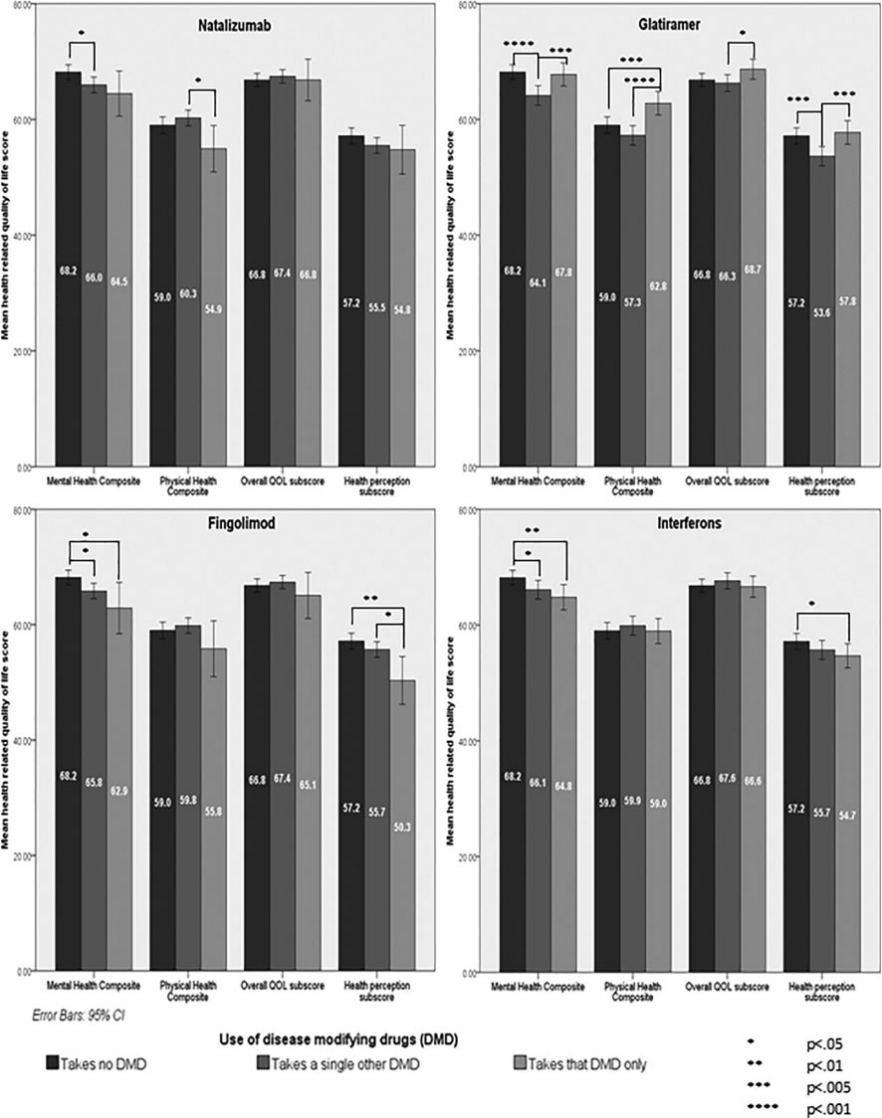
Health-related quality of life (HRQOL) by individual DMDs, all other DMDs and no DMD use

There were trends, some statistically significant, as shown in Fig. 1, to worse HRQOL across the four domains (mental health composite, physical health composite, overall QOL subscore and health perception subscore) for the interferons, fingolimod and natalizumab when compared with the other DMDs and more so when compared with no DMD use. In contrast, glatiramer acetate showed favourable trends for HRQOL when compared with the other DMDs across these domains and was the only medication for which HRQOL was higher in any of the domains than for those taking no DMDs. Overall, the size of these differences, however, was small and unlikely to be of clinical significance despite being statistically significant.

**Figure 2.**
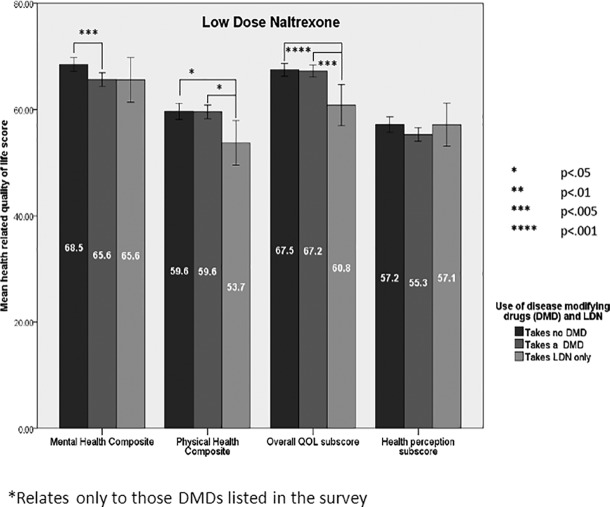
Health-related quality of life (HRQOL) for low-dose naltrexone (LDN), all other DMDs and no DMD use*

Low-dose naltrexone was frequently being used in addition to one of the first or second generation DMDs. Of 163 people taking LDN, 32 were also taking glatiramer, 9 interferons, 4 fingolimod, 2 dimethyl fumarate, 2 natalizumab, 1 teriflunomide and 1 alemtuzumab. In addition, eight people taking LDN were also taking minocycline. The HRQOL for LDN was significantly lower across many domains than for those taking ‘other DMDs’ and those not taking DMDs (Fig. 2).

Head-to-head comparisons between each of the four most commonly used DMDs and against no DMD use for each of the domains of the MSQOL-54 where significant differences were found are shown in Table 5. Glatiramer acetate was associated with better QOL than the other DMDs across most domains.

**Table 5:** HRQOL composites and subscores comparing each of the four most frequently used DMDs against no DMD use

HRQOL composite/subscore	DMD used	*n* =	Mean HRQOL	SD	*P*	Pairwise comparisons*
Physical health composite	1. Natalizumab only	107	54.9	20.9	**0.002**	3 vs 1, *P* = 0.001
	2. Fingolimod only	71	55.8	20.5		3 vs 2, *P* = 0.012
	3. Glatiramer only	408	62.8	20.8		3 vs 4, *P* = 0.014
	4. Interferons only	351	59.0	20.7		3 vs 5, *P* = 0.003
	5. No DMD	919	59.0	22.1		
Mental health composite	1. Natalizumab only	118	64.5	21.4	**0.012**	5 vs 2, *P* = 0.027
	2. Fingolimod only	85	62.9	20.6		5 vs 4, *P* = 0.007
	3. Glatiramer only	450	67.8	21.7		3 vs 4, *P* = 0.045
	4. Interferons only	384	64.8	21.7		
	5. No DMD	1078	68.2	21.0		
Physical health subscore	1.Natalizumab only	122	56.6	32.7	** < 0.001**	3 vs 1, *P* < 0.001
	2. Fingolimod only	86	55.8	33.6		3 vs 2, *P* = 0.001
	3. Glatiramer only	463	68.6	30.0		3 vs 4, *P* = 0.013
	4. Interferons only	401	63.1	30.5		3 vs 5, *P* < 0.001
	5. No DMD	1125	56.4	34.9		4 vs 5, *P* < 0.001
Role limitations physical subscore	1. Natalizumab only	119	43.1	43.8	**0.023**	3 vs 1, *P* = 0.044
	2. Fingolimod only	86	44.2	42.1		3 vs 4, *P* = 0.034
	3. Glatiramer only	463	52.0	43.8		3 vs 5, *P* = 0.001
	4. Interferons only	400	45.8	43.1		
	5. No DMD	1123	44.3	43.3		
Pain subscore	1. Natalizumab only	121	70.4	27.9	**0.003**	3 vs 2, *P* = 0.001
	2. Fingolimod only	85	65.6	27.4		3 vs 4, *P* = 0.003
	3. Glatiramer only	463	75.5	24.8		3 vs 5, *P* = 0.017
	4. Interferons only	397	70.1	26.2		2 vs 5, *P* = 0.029
	5. No DMD	1129	72.0	26.2		
Emotional wellbeing subscore	1. Natalizumab only	122	67.1	19.4	**0.006**	4 vs 5, *P* < 0.001
	2. Fingolimod only	86	67.6	18.7		
	3. Glatiramer only	464	68.6	19.0		
	4. Interferons only	402	66.3	19.0		
	5. No DMD	1132	70.1	18.2		
Health perception subscore	1. Natalizumab only	122	54.8	23.5	**0.017**	3 vs 2, *P* = 0.005
	2. Fingolimod only	86	50.3	19.3		3 vs 4, *P* = 0.047
	3. Glatiramer only	464	57.8	22.3		5 vs 2, *P* = 0.007
	4. Interferons only	401	54.7	21.4		
	5. No DMD	1130	57.2	23.4		
Social function subscore	1. Natalizumab only	117	66.7	22.8	**0.005**	3 vs 1, *P* = 0.006
	2. Fingolimod only	83	68.1	22.6		3 vs 4, *P* = 0.008
	3. Glatiramer only	455	73.6	22.6		3 vs 5, *P* = 0.001
	4. Interferons only	388	69.1	24.0		
	5. No DMD	1094	69.0	24.9		
Cognitive function subscore	1.Natalizumab only	122	63.4	27.9	**0.008**	5 vs 2, *P* = 0.004
	2. Fingolimod only	86	59.5	29.1		5 vs 4, *P* = 0.038
	3. Glatiramer only	464	65.5	27.2		
	4. Interferons only	402	65.0	27.2		
	5. No DMD	1132	68.2	26.3		
Health distress subscore	1.Natalizumab only	122	56.8	28.0	**0.033**	4 vs 5, *P* = 0.027
	2. Fingolimod only	86	55.6	27.5		
	3. Glatiramer only	464	61.0	27.6		
	4. Interferons only	402	58.3	26.8		
	5. No DMD	1130	61.8	27.0		
Sexual function subscore	1. Natalizumab only	113	64.2	31.5	**0.033**	3 vs 2, *P* = 0.035
	2. Fingolimod only	74	62.4	29.2		3 vs 4, *P* = 0.039
	3. Glatiramer only	422	70.5	29.4		3 vs 5, *P* = 0.004
	4. Interferons only	369	66.0	30.2		
	5. No DMD	976	65.4	31.3		

Only domains with significant differences are shown.

HRQOL: health-related quality of life; DMD: disease-modifying drug.

* Pairwise comparison with bonferroni adjustment applied, significant differences only shown. Bold denotes statistically significant.

Regression analyses revealed that taking a single DMD was associated with a statistically significant but only slight (2.3 point, 95% CI 0.5–4.1) reduction on the mental health composite score HRQOL but not on the physical health composite score. However, after controlling for a range of relatively stable factors (age, marital status, employment status, education, number of close relationships, disability and number of comorbidities), small significant associations were found for both outcomes; use of a single DMD was associated with a 1.9 (95% CI 0.5–3.3, *P* = 0.007) point reduction on the physical health composite score and a 1.6-point reduction (95% CI 0.1–3.2, *P* = 0.049) in mental health composite score.

#### Health-related quality of life and polypharmacy

Respondents taking five or more over the counter, prescription or herbal agents, irrespective of DMD use, scored statistically significantly lower for every domain of HRQOL than those not reporting polypharmacy use, with differences in HRQOL scores ranging between 9.9–31.5 points depending on domain (results not shown). For every domain of HRQOL, this pattern was also present for the subsample taking a single DMD for ≥ 12 months and those not taking a DMD (data not shown).

#### Relapse rate

Overall, 12 month specialist-diagnosed relapse rate was comparable for those taking a single DMD (0.73/year, *n* = 840) compared with those not taking a DMD (0.67/year, *n* = 491) (*P* = 0.146); however, the subset of participants taking a single DMD for over 12 months had a significantly lower relapse rate (0.51/year, *n* = 562) than those not taking a DMD (0.67/year, *n* = 491) (*P* = .006), a 23.9% lower relapse rate. There were no significant differences in relapse rates associated with each of the individual major DMDs (glatiramer, interferons, fingolimod and natalizumab) when compared with relapse rates for those taking any other single DMD, or with relapse rates for those not taking any DMD, when corrected for multiple comparisons, and for those taking a single DMD longer than 12 months. Relapse rate was significantly higher (56.1%) among respondents with polypharmacy (1.03/year, *n* = 179) than those without (0.66/year, *n* = 1178) (*P* = 0.005). This pattern was present among those with ≥ 12 months single DMD use (polypharmacy: 0.90/year, *n* = 86 vs no polypharmacy: 0.44/year, *n* = 476, *P* = 0.03), and also those not taking a DMD (polypharmacy: 1.19/year, *n* = 47 vs no polypharmacy: 0.62/year, *n* = 444, *P* < 0.001).

#### Disability

People with multiple sclerosis using a single DMD were over-represented in the group with normal/some disability and under-represented in major disability (Table 6). There was no significant difference in disability level, however, for the subset taking a single DMD for over 12 months.

**Table 6:** Number and percentage of PwMS in each major disability category by DMD use and polypharmacy

		Level of disability (PDDS)				
		Normal/some *n* (%)	Moderate *n* (%)	Major *n* (%)	Total *n* (%)	*P*
DMD use	Yes	674 (61.6)*	355 (32.4)	66 (6.0)†	1095 (100)	< 0.001
	No	557 (49.2)†	410 (36.2)	166 (14.7)*	1133 (100)	
	Total	1231 (55.3)	765 (34.3)	232 (10.4)	2228 (100)	
Polypharmacy	Yes	107 (31.6)†	165 (48.7)*	67 (19.8)*	339 (100%)	< 0.001
	No	1160 (59.2)*	628 (32.0)†	173 (8.8)†	1961 (100%)	
	Total	1267 (55.1)	793 (34.5)	240 (10.4)	2300 (100)	
Polypharmacy among subsample with DMD use	Yes	48 (41.4) †	56 (48.3)*	12 (10.3)*	116 (100.0)	< 0.001
	No	393 (64.3)*	185 (30.3) †	33 (5.4) †	611 (100.0)	
	Total	441 (60.7)	241 (33.1)	45 (6.2)	727 (100.0)	
Polypharmacy among subsample without DMD use	Yes	38 (24.7)†	70 (45.5)*	46 (29.9)*	154 (100.0)	< 0.001
	No	519 (53.0)*	340 (34.7%)†	120 (12.3%)†	979 (100.0)	
	Total	557 (49.2)	410 (36.2)	166 (14.7)	1133 (100.0)	

DMD: disease-modifying drug; PDDS: patient-determined disease steps; PwMS: people with multiple sclerosis.

* Bold faces denote significantly over-represented as determined by standardized adjusted residuals.

† Denotes significantly under-represented as determined by standardized adjusted residuals.

Overall, those taking glatiramer for any length of time were significantly over-represented among those with normal/some disability, and those taking natalizumab were significantly under-represented in this disability category and significantly over-represented among those with major mobility impairment (*P* = .031). Again, however, these differences were not apparent among respondents taking a DMD for >12 months. Those with polypharmacy, irrespective of DMD use, were significantly over-represented in the more disabled categories (Table 6). This pattern remained when the subsamples of those with and without DMD use for >12 months were analysed separately but was of greater magnitude among those without DMD use (Table 6).

## Discussion

### Sample characteristics and opportunities

Our data represent a unique snapshot of the medication use of ∼2500 PwMS worldwide. Probably by virtue of our sampling of Web 2.0 platforms and forums, our cohort comprised a greater than usual ratio of women to men (4.5 : 1), with likely relatively good premorbid health as judged by 58% of our sample, from predominantly western countries, having normal or low BMI. This is nearly double the rate of those with a normal or low BMI in the US general population, the country most represented in our sample, where only 31% of the population had a normal or low BMI in 2011–12.^24^ With about half the sample taking one of the DMDs, this provided an opportunity to compare commonly measured disease outcomes by medication use and by particular medications. With most medication studies funded by the pharmaceutical industry, few independent studies of similar scale to our study have addressed the challenging issue of assessing real-world DMD associations with HRQOL, disease activity and disability. A large prospective Italian observational study found earlier institution of interferon beta significantly reduced the risk of progression^25^ however, this was not confirmed by a more recent Canadian study which found that interferon beta administration was not associated with reduced disability.^26^ Both studies had the advantage over our study of availability of disability data over time. Planned longitudinal follow-up of our cohort may enable better understanding of cause and effect of the associations observed in our study.

### Medication use

The pattern of medication use by PwMS in our sample provided some unique perspectives on their medication choices and the prescribing habits of their clinicians. The proportion of participants taking DMDs was relatively low, probably influenced by the fact that many patients came from countries where DMDs are not reimbursed by healthcare systems. First generation self-injected DMDs were still the most commonly used medications in our sample, with a significant proportion of respondents having taken them for longer than a decade, although most had taken them for between 1 and 10 years. Doctors treating PwMS are still prescribing these medications commonly, despite the arrival of a range of oral DMDs, as around a quarter of those taking these medicines had been taking them for less than a year. This may be contributed to by the fact that, in some countries, the newer oral agents are not subsidized by government and may be cost prohibitive. Natalizumab was also frequently prescribed; however, many people had taken the drug for some years, raising concerns about the potential for the development of progressive multi-focal leukoencephalopathy (PML), known to occur more frequently with prolonged use.^1^ Fingolimod was used more frequently than the other approved oral medications reflecting its earlier licensing approval in most countries. It was interesting to note how commonly LDN was used in this cohort, despite not being licensed for use in MS in any country. There is some evidence from randomized controlled trials of a possible benefit for HRQOL with this medication^27^; however, the authors detected no positive associations of LDN with HRQOL or relapse rate in our sample.

Despite a lack of evidence for any benefit in MS, several immunosuppressants were used for a small proportion of the sample. Similarly, while steroids had been used in the past in ∼30% of our sample, presumably for the management of relapses, and ∼6% were currently taking a steroid, it was of some concern that a number of PwMS appeared to be taking these medications long term. Previous studies have shown no benefit from long term use of steroids in MS, with the risk of serious side effects.^28^ Many PwMS in our study were taking the symptom modifying drugs baclofen for spasticity and fampridine to improve walking, although around half as many had stopped taking these medications, presumably because of side effect concerns or lack of efficacy.

It is well known that many PwMS choose to discontinue their DMDs.^29^ In our study, ∼15% had ceased a previous DMD and not taken an alternative medication, and a slightly larger proportion had replaced a previous DMD with another medication. Mostly, this involved people ceasing one of the interferons (nearly a quarter of the sample). This is likely to reflect side effects, known to be more common with the interferons than the other DMDs. Data from this cohort also suggest a negative effect on mood,^30^ and this may have contributed to discontinuation of the drug. It probably also reflects preferences for oral agents over injectable drugs.

A large proportion of PwMS in our sample were taking over the counter, prescription and herbal agents such as paracetamol, St John's Wort and magnesium, for the common symptoms that accompany MS, particularly depression, pain and spasticity. Many were taking a large number of such agents, raising the issue of drug interactions and side effects. Similarly, a large proportion were taking medication for fatigue, despite a lack of evidence for any benefit from such medications.^31^ This contrasts with the strong associations found between healthy lifestyle choices and reduced fatigue in this same cohort described elsewhere.^32^ Given previous data suggesting worse fatigue and cognitive deficit in those PwMS on multiple pharmacological agents,^33^ the extent of polypharmacy in this cohort was of concern, with over one-third of the cohort taking three or more over the counter, prescription or herbal agents for symptom management, and ∼15% taking five or more, excluding their use of DMDs.

### Medication use and disease outcomes

Our study represented an opportunity to observe, in a real-world situation, over 56 different countries, the association of a variety of disease outcomes with medication use and a comparison between different medications for these outcomes. Our lack of data on these outcomes before exposure to DMDs limits extrapolation of the cross-sectional associations to potential temporal relationships, or inferences of cause and effect. It is likely that prescription of DMDs, changes from one DMD to another and stopping a previously prescribed DMD are in many cases the result of disease activity and disability level. In turn, QOL would be expected to be directly affected by disease activity and disability level. Thus, there are significant issues of reverse causation and confounding by indication, which limit the conclusions that can be drawn from our data.

Nonetheless, our cross-sectional data represent a unique snapshot of medication use from a geographically diverse population and allow examination of the association between medication use and health outcomes important to PwMS and their clinicians. Quality of life outcomes have previously been highlighted as an unmet need in current MS management.^34^,^35^ Our study revealed no real favourable pattern of association of DMD use with HRQOL outcomes, with only 6 of 14 domains showing statistically significant associations with medication use, 2 favourably, and 4 in favour of those not using the DMDs. The differences were however very small, with only one domain, the physical health subscore showing a favourable *clinically* significant association with DMD use. Generally, across all DMDs, there was no particular indication of any significant association with QOL. Regression analysis revealed essentially insignificant QOL associations with medication use.

Of the DMDs, only glatiramer acetate was positively associated with HRQOL, with the magnitude small but bordering on clinically significant. These marginally positive QOL associations are in keeping with previous literature^36^ and fit with recent data on 672 PwMS from 148 centres worldwide, showing improvements in health outcomes including QOL for those switching from other medications to glatiramer.^37^ While glatiramer appeared to be associated with better QOL for PwMS, compared with other medications, this may reflect its prescription for people with less aggressive disease.

While there was no difference in relapse rates for those people with RRMS taking one of the four major DMDs compared with those not, or taking any other DMD, for those on a DMD >12 months, there was a small but significant reduction in doctor-diagnosed relapse rate from 0.67/year to 0.51/year, a 24% reduction. For disability, those taking a DMD >12 months did not differ significantly in disability from those not taking a DMD. The authors did not detect any signal of an association of DMD use with disability reduction in those on longer term DMDs, although the lack of longitudinal data on disease outcomes precludes any meaningful conclusion from this finding. The authors confirmed previous concerns about polypharmacy for the QOL of PwMS^33^ and raise concerns about higher relapse rates and more disability.

Future research into efficacy of DMDs for PwMS should include measures of HRQOL. Long term population studies with longitudinal data on medication use, QOL, relapse rate and disability are required to better understand the efficacy of these medications in the MS population. Our planned longitudinal follow up should help clarify these questions.

### Limitations

All data in our study were self-reported. The authors were therefore unable to verify medication use, disease type or relapse rates. Our novel recruitment using social media enabled us to access considerably more PwMS than many other studies and may be considered by other researchers wishing to examine factors affecting the health of PwMS. This very large sample size, to some extent, balances the limitations of reduced data reliability because of self-report. Our data were observational and cross-sectional, and hence cannot prove cause and effect. Without baseline data on disease activity and disability before initiating DMDs, there is no reliable means of telling whether DMDs influence these outcomes or whether people have taken DMDs because of disease activity, disability or both. Quality of life in turn would be expected to be affected by disease activity and disability. This may have affected our observed associations. Our data were from English-speaking participants of 75 different countries of birth residing in 56 different countries and therefore should generalize broadly.

## Conclusion

Our real-world snapshot of self-reported medication use by a large sample of PwMS worldwide detected a signal for those taking a single DMD for >12 months of the relapse rate reductions reported in clinical trials, but demonstrated no particular association with disability, and inconsistent and generally minor associations with HRQOL. Glatiramer may have some advantages for HRQOL over other DMDs, including newer generation medications, although reverse causality may have been a factor in this association. Polypharmacy for people with MS was associated with considerably poorer health and QOL.

## Disclaimer Statements

**Contributors** GJ, principal and corresponding author, conceived and supervised the study, drafted the manuscript, was involved in data analysis and interpretation and gave final approval for submission. TW helped revise the manuscript, was principally responsible for data analysis and gave final approval for submission. EH helped revise the manuscript, was principally responsible for data collection, was involved in data analysis and gave final approval for submission. CM helped revise the manuscript, assisted with data collection, was involved in data analysis and gave final approval for submission. NP helped revise the manuscript, was involved in accessing the validated tools for the study and gave final approval for submission. DM helped revise the manuscript, was involved in data analysis and gave final approval for submission.

**Funding** The authors are grateful for the philanthropic support of the Bloom Foundation, the Horne Family Charitable Trust, and fundraising donations from Elizabeth Schefferle.

**Conflicts of interest** Prof. GJ receives royalties for his book ‘Overcoming Multiple Sclerosis: An Evidence-Based Guide to Recovery published by Allen and Unwin’.

**Ethics approval** This study was approved by the Human Research Ethics Committee at St Vincent's Hospital, Melbourne, Australia.
